# Isolation, Identification, and Antimicrobial Susceptibility of Bacteria Associated with Waterpipe Contaminants in Selected Area of Saudi Arabia

**DOI:** 10.1155/2017/8042603

**Published:** 2017-08-28

**Authors:** Mohammed Alaidarous, Meshal Alanazi, Ahmed Abdel-Hadi

**Affiliations:** Department of Medical Laboratory Sciences, College of Applied Medical Sciences, Majmaah University, Majmaah, Riyadh, Saudi Arabia

## Abstract

This study highlights the level of microbial contamination of waterpipe components in selected area of Saudi Arabia and the resistance of selected bacteria to different antibiotics was determined. A series of biochemical tests, microscopic examination, and screening on Vitek 2 compact (bioMérieux Inc., USA) system were done to characterize the bacterial isolates. Out of 132 samples investigated, 7 mouthpiece samples and 48 water bowl samples showed positivity on culture. The percentage of contamination rate was higher in water bowl (69.69%) than in mouthpieces (10.6%) for all selected areas. A total of 55 bacterial isolates were identified which included Gram-negative (28) and Gram-positive (27) bacteria. Antimicrobial susceptibility data showed more resistance to bacteria isolated from water bowl than bacteria isolated from mouthpiece. In addition, one isolate which was confirmed as methicillin-resistant* Staphylococcus aureus* and* Klebsiella pneumoniae* was resistant to antibiotics which are commonly used to treat pneumonia. Water bowl of waterpipe instrument is significantly contaminated with different bacterial pathogens including multidrug-resistant and pneumonia causing bacteria, which are a real health concern among waterpipe smokers. The presented data could assist public health professionals to raise the concerns regarding cleaning practices of waterpipe components and highlights the risk posed among the waterpipe smokers.

## 1. Introduction

**Figure 1:**
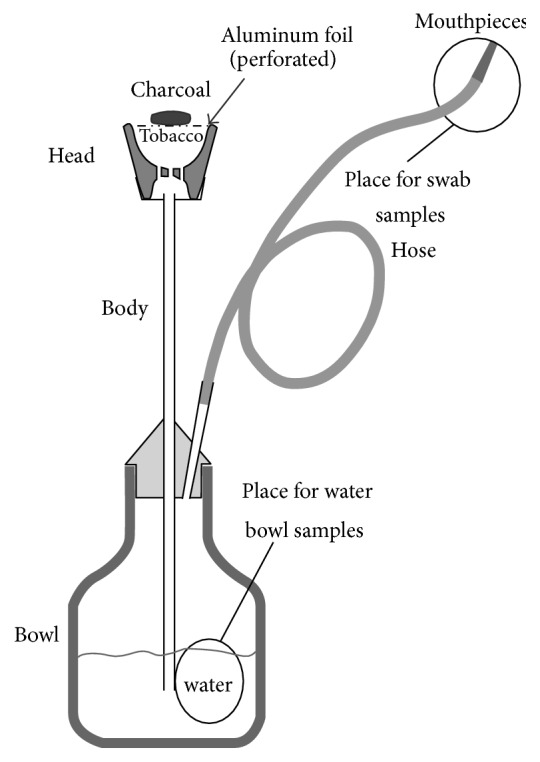
Schematic of a narghile waterpipe (Monzer et al. [2]).

Waterpipe smoking (also known as narghile, shisha, hubbly bubbly, gozza, boori, or hookah) has been widely used to smoke tobacco in Africa and Asia as a cultural phenomenon [1]. Narghile waterpipe as shown in Figure 1 is an instrument used to smoke tobacco where in the smoke passes through water before the inhalation by the users. A heavily sweetened and flavored tobacco mixture (called Mo'assal) is loaded into the head and is burned by lit charcoal. When a user sucks through the hose via the mouthpiece, a vacuum is produced in the space above the waterline, causing smoke to bubble into the water bowl from the body. A disposable plastic tips may be offered for the users to be attached to the fixed mouthpiece at the end of the hose [2]. Recently, the use of the waterpipe has become a social phenomenon as with cigarette smoking, with hookah bars, cafés, and restaurants becoming popular social gathering places for young smokers [3]. According to the study that assessed the smoking habits in ten countries in the Middle East and North Africa especially in Saudi Arabia, the most frequent type of smoking was found to be waterpipe [4].

It is obvious that there are risks of spreading microbial infections with commensal and pathogenic microbes through waterpipe smoking. Most frequently, as several people use waterpipe simultaneously, the moist nature of waterpipe molasses creates conducive environment for the growth of various microorganisms. Although some individuals regularly wash their waterpipes, the relatively rigid and complicated structure of the waterpipe makes it virtually impossible to efficient washing of all its components, and for that reason different types of pathogenic bacteria may grow and survive on the internal surface of the waterpipe [5].

According to Munckhof et al., [6] cases of patients with pulmonary tuberculosis (TB) were documented in Queensland, Australia, as a result of sharing a marijuana waterpipe with a case of pulmonary tuberculosis. In the Middle East, outbreaks of infectious disease have been correlated with shisha smoking. Akl et al. [7] described two outbreaks in 2010, which revealed a possible association between TB and sharing a shisha pipe.

Hence, the present study was designed to identify the bacteria that contaminate waterpipe components used in cafés of selected area in the Kingdom of Saudi Arabia and to investigate the drug resistance of the isolated bacteria to various antibiotics.

## 2. Methods

### 2.1. Sample Collection

This study was performed in 3 cities of Saudi Arabia: Riyadh, the capital city of Saudi Arabia, Al-Ghat, a town in Riyadh Province, and Hafar Al-Batin, city in the Eastern Province. Ten waterpipe cafés in different parts of Riyadh (3 cafés), Al-Ghat (2 cafés), and Hafar Al-Batin (5 cafés) were randomly selected. Samples were collected from the water of bowl and fixed mouthpiece of the waterpipe. For collection of water samples of the bowl, 10 ml water was taken using a sterile syringe and placed into sterile bottle. The owners reported that the water used in the bowl is a drinking water and changing of water is applied daily. Mouthpiece samples were collected by swabbing the top portion of the internal waterpipe hose using the BD BBL™ culture swab™ collection and transport systems. During sampling processes, aseptic practices were followed. A total of 132 samples, 30 samples from Riyadh (15 swabs and 15 water bowls), 60 samples from Al-Ghat (30 swabs and 30 water bowls), and 42 samples from Hafar Al-Batin (21 swabs and 21 water bowls), were collected.

### 2.2. Bacterial Isolation and Characterization

The collected samples were inoculated on Nutrient agar, blood agar, and MacConkey agar and incubated at 37°C for 48 hours. After the incubation period, the isolated colonies were further subjected to purification and subculture. Nutrient agar, 5% sheep blood agar, was used to select the pure colonies of the isolates. Preliminary identification of the each isolate was done using gram stain, catalase, and oxidase. Subsequently, selective media such as Eosin methylene blue agar (EMB) was used if the isolates were suspected as* E. coli*.

### 2.3. Bacterial Identification

Identification was performed with the Vitek 2 compact (bioMérieux Inc. USA) system using GP ID REF21342 (identification-Gram-positive bacteria) and GN ID REF21341 (identification-Gram-negative bacteria) cards. All the test procedures were followed according to the manufacturer's instructions.

### 2.4. Antimicrobial Susceptibility Testing

AST-P580 (*Staphylococcus* spp.,* Enterococcus* spp., and* S. agalactiae*), AST-P506 (pneumococci), and AST-N291 (Gram-negative bacilli) cards were used to determine antibiotic susceptibility and the results were interpreted using Vitek 2 compact software version 07.01.

## 3. Results

**Figure 2:**
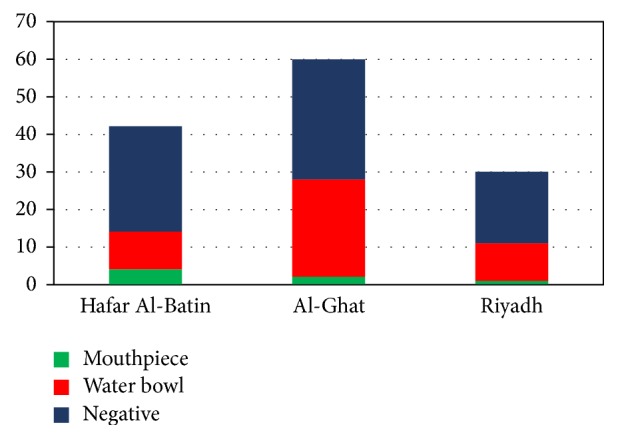
Bacterial frequency in collected samples from waterpipe components.

The results in Figure 2 indicates that 46 (69.69%) of 66 samples from water bowl and 7 (10.6%) of 66 samples from mouthpiece were positive in the three selected areas. The percentage of contamination was high in Al-Ghat area, where 26 (86.6%) of 30 samples from water bowl and 2 (6.6%) of 30 samples from mouthpiece were contaminated. In Riyadh area, the percentage of contamination was 66.6% (10 out 15 samples) from water bowl and 6.6% (1 out of 15 samples) from mouthpiece. In Hafar Al-Batin area, 10 (47.6%) of 21 samples from water bowl and 4 (19%) of 21 samples from mouthpiece were contaminated.

Total of 55 bacterial isolates belonging to 19 different species were identified as waterpipe contaminants. 51% of the total isolates are comprised of 11 different species of Gram-negative bacteria, while 49% of the total isolates are comprised of 8 different species Gram-positive bacteria. 48 isolates belonging to 17 species were isolated from water bowl, while only 7 isolates belonging to five strains were isolated from mouthpiece. Hafar Al-Batin and Riyadh samples were seen to have higher contamination of Gram-negative than Gram-positive bacteria, compared to Al-Ghat samples which were more contaminated with Gram-positive than Gram-negative bacteria. In Riyadh 7 Gram-negative bacteria,* Escherichia coli*,* Klebsiella oxytoca*,* Comamonas testosteroni*,* Enterobacter cloacae complex*,* Stenotrophomonas maltophilia*,* Bordetella bronchiseptica*, and* Cupriavidus pauculus*, and two Gram-positive bacteria, belonging to* Kocuria rhizophila* and* Kocuria kristinae,* were identified. In Hafar Al-Batin, 5 Gram-negative bacteria,* Klebsiella oxytoca*,* Pseudomonas putida*,* Sphingomonas paucimobilis*,* Pseudomonas aeruginosa*, and* Klebsiella pneumoniae*, and one Gram-positive bacterium,* Aerococcus viridans, *were identified. Similarly, in Al-Ghat, 6 Gram-positive bacteria belonging to* Staphylococcus aureus*,* Streptococcus pneumonia*,* Staphylococcus vitulinus*,* Streptococcus thoraltensis*,* Kocuria rhizophila*, and* Kocuria rosea* and 3 Gram-negative bacteria belonging to* Enterobacter cloacae complex*,* Klebsiella pneumoniae*, and* Pseudomonas aeruginosa* were identified (Tables 1 and 2). The most frequently isolated organisms were* Kocuria rhizophila* (10 isolates) followed by* Enterobacter cloacae* complex (8 isolates) and* Staphylococcus aureus* (5 isolates).

Six Gram-negative bacteria were selected for antimicrobial susceptibility including 3 isolates from Al- Ghat water bowl* (Klebsiella pneumoniae*,* Enterobacter cloacae complex*, and* Pseudomonas aeruginosa)*, 1 isolate from Hafar Al-Batin water bowl* (Pseudomonas putida)*, 1 isolate from Riyadh mouthpiece* (Escherichia coli)*, and 1 isolate from Hafar Al-Batin mouthpiece* (Klebsiella oxytoca)*. Another 6 Gram-positive bacteria including 3 isolates from Al-Ghat water bowl* (Staphylococcus aureus*,* Staphylococcus vitulinus*, and* Streptococcus pneumonia)*, 1 isolate from Hafar Al-Batin mouthpiece* (Aerococcus viridians)*, 1 isolate from Al-Ghat mouthpiece* (Streptococcus pneumonia)*, and 1 isolate from Riyadh mouthpiece* (Kocuria rhizophila)* were examined for their susceptibility to wide range of antibiotics.

Our antimicrobial susceptibility results (Table 3) indicate that* Klebsiella pneumoniae*,* Pseudomonas putida*, and* Pseudomonas aeruginosa* exhibited a more resistant to several antimicrobial agents than the other Gram-negative tested bacteria. Resistance to ampicillin, amoxicillin/clavulanic acid, cefoxitin, nitrofurantoin, tigecycline, and trimethoprim/sulfamethoxazole was observed.  Table 4 indicates that* Staphylococcus aureus*,* Staphylococcus vitulinus*, and* Streptococcus thoraltensis *were positive to cefoxitin screen and resistant to benzylpenicillin, oxacillin, erythromycin, clindamycin, and trimethoprim/sulfamethoxazole. In addition,* Streptococcus pneumonia *showed resistance to benzylpenicillin and erythromycin.

## 4. Discussion

To the best of our knowledge, this was the first study in Saudi Arabia to investigate the microbial contamination of waterpipe components that can transmit the infection to the smokers. The present study revealed bacterial contamination of mouthpieces and water bowl with several pathogenic bacteria and waterborne bacteria that can act as pathogens. Generally, the percentage of contamination was higher in water bowl (69.69%) than in mouthpieces (10.6%) for all three selected areas. Al-Ghat area showed higher contamination level followed by Riyadh and Hafar Al-Batin. This could be due to regular change of water bowl and cleaning practices in big cities (Riyadh and Hafar Al-Batin) compared to the smaller town of Al-Ghat. In contrast to our findings, Safizadeh et al. [8] have shown higher level of contamination in both water bowl (96%) and fixed mouthpiece (69%). This could be due to fact that they collected more number of samples (285) from one area compared to our 132 samples collected from three different areas. Also, the part for swab collection was different, where they collected the samples from the bottom of hose, which is near to water bowl, while our samples were collected from the top of hose which is far from water bowl.

The present study showed bacterial contamination of water bowl with Gram-negative bacteria including* Escherichia coli *in Riyadh*, Klebsiella pneumoniae*, and* Pseudomonas aeruginosa* in Al-Ghat and Hafar Al-Batin,* Klebsiella oxytoca *in Riyadh and Hafar Al-Batin, and* Enterobacter cloacae complex* in Al-Ghat and Riyadh. Gram-positive bacteria including* Staphylococcus aureus*,* Staphylococcus vitulinus*, and* Streptococcus thoraltensis* were recorded as water bowl contaminants in Al-Ghat. It was reported that the most common organisms in skin and skin structure infections include* Staphylococcus aureus, Pseudomonas aeruginosa*,* Escherichia coli*,* Klebsiella pneumoniae*, and* Enterobacter cloacae* [9]. This suggests that the presence of these contaminants can be from water subjected to human interaction during cleaning practices. Water bowl can be oligotrophic environments with sufficient nutrients to maintain bacterial growth and releasing organic matter from smoking can provide additional substrates for the microbial growth. Daniels and Roman [10] documented that the moist of the tobacco creates conditions for the development and growth of various microorganisms. Most of the isolated bacteria are included among the potential causes of bacterial pneumonia and infections in lower respiratory tract [11–13]. Recently, Mahmoud et al. [14] reported that tobacco tar increases microbial hydrophobicity that leads to the increase of microbial adherence to epithelial cells and their colonization, which is considered the first step in developing invasive infections. Interestingly, this study has shown that* Klebsiella oxytoca* and* Aerococcus viridians* contaminated water bowl and mouthpiece of the same samples in Hafar Al-Batin and* Kocuria rhizophila* in Al-Ghat and Riyadh. This can be attributed to the possibility of carrying bacteria from contaminated water bowl to mouthpiece through smoke and then can be transmitted to the smokers. Interestingly,* Streptococcus pneumonia* and* Sphingomonas paucimobilis* were only recorded as mouthpiece contaminants. This can be attributed to the possibility of transmission of these bacteria from the users to mouthpiece. It was reported that* Streptococcus pneumonia* and* Sphingomonas paucimobilis* could cause oral infection [15, 16]. The potential existence of mycobacteria in the narghile tube as a source of contamination of different smokers was recorded [17].

Antibiotic susceptibility test of the isolates indicates the presence significant levels of resistance to antibiotics for bacteria isolated from water bowl in comparison to bacteria isolated from mouthpiece. This indicates that water bowl can create a type of resistance for bacteria contaminating it. Interestingly,* Staphylococcus aureus* was positive to cefoxitin screening and showed high level resistance to wide range of antibiotics including oxacillin. This isolate was further confirmed as methicillin-resistant* Staphylococcus aureus* (MRSA). Cefoxitin susceptibility testing has greatly improved the reliability of detecting methicillin resistance* Staphylococcus aureus* [18]. Recently, McEachern et al. [19] reported that MRSA induced upregulation of mprF gene after being exposed to cigarette smoke extract. This leading to surface charge modifications and MRSA can become more resistant to human antimicrobial peptide.

## 5. Conclusion 

Our study highlighted the level of microbial contamination in waterpipe components with different pathogenic bacteria which varied from region to region in the Kingdom of Saudi Arabia. Water bowl can act as a good environment for the growth of bacterial pathogens and also the microbes can acquire/develop drug resistance capability [14]. Smoke which is drawn through water can transmit bacterial pathogens to the smokers and cause serious health effects. More studies on the effect of chemical emitted from tobacco on antimicrobial susceptibility of detected bacteria are warranted. Based on the presented data, there is a significant risk of transmitting multidrug-resistant bacteria among waterpipe smokers, which is a serious health problem in these regions. There is a limitation in our study that control samples were not planned, as the study was initiated with small number of samples. However, a larger study is planned in near future to overcome the above-mentioned limitation. Larger studies with multiple locations are needed to investigate the situation, which will surely help the public health authority to prevent any waterpipe associated disease outbreaks.

## Figures and Tables

**Table 1:** Frequency of Gram-negative bacteria isolated from waterpipe components, where M refers to mouthpiece and W to water bowl.

Bacteria	Riyadh		Hafar Al-Batin		Al-Ghat		Total
	M	W	M	W	M	W	
*Escherichia coli*	—	1	—	—	—	—	1
*Klebsiella oxytoca *	—	1	1	2	—	—	4
*Klebsiella pneumoniae*	—	—	—	1	—	2	3
*Pseudomonas putida*	—	—	—	3	—	—	3
*Pseudomonas aeruginosa*	—	—	—	2	—	1	3
*Sphingomonas paucimobilis*	—	—	2	—	—	—	2
*Comamonas testosterone*	—	1	—	—	—	—	1
*Enterobacter cloacae complex*	—	1	—	—	—	7	8
*Stenotrophomonas maltophilia*	—	1	—	—	—	—	1
*Bordetella bronchiseptica*	—	1	—	—	—	—	1
*Cupriavidus pauculus*	—	1	—	—	—	—	1

Total	—	7	3	8	—	10	28

**Table 2:** Frequency of Gram-positive bacteria isolated from waterpipe components where M refers to mouthpiece and W to water bowl.

Bacteria	Riyadh		Hafar Al-Batin		Al-Ghat		Total
	M	W	M	W	M	W	
*Staphylococcus aureus*	—	—	—	—	—	5	5
*Staphylococcus vitulinus*	—	—	—	—	—	1	1
*Streptococcus pneumonia*	—	—	—	—	1	—	1
*Streptococcus thoraltensis *	—	—	—	—	—	2	2
*Aerococcus viridians*	—	—	1	1	—	—	2
*Kocuria rhizophila*	1	1	—	—	1	7	10
*Kocuria rosea*	—	—	—	—	—	4	4
*Kocuria kristinae*	—	2	—	—	—	—	2

Total	1	3	1	1	2	19	27

**Table 3:** Antibiotics susceptibility against selected Gram-negative bacteria.

Antimicrobial agents	Bacteria											
	*Escherichia coli*		*Klebsiella oxytoca*		*Klebsiella pneumoniae*		*Pseudomonas aeruginosa*		*Pseudomonas putida*		*Enterobacter cloacae *complex	
	MIC	Inter	MIC	Inter	MIC	Inter	MIC	Inter	MIC	Inter	MIC	Inter
ESBL	NEG	—	NEG	—	NEG	—	NEG	—	NEG	—	NEG	—
Ampicillin	≥32	R	≥32	R	≥32	R	≥32	R	≥32	R	16	I
Amoxicillin/clavulanic acid	16	I	≤2	S	≥32	R	≥32	R	≥32	R	≤2	R^*∗*^
Piperacillin/tazobactam	64	I	≤4	S	16	R^*∗*^	8	I^*∗*^	32	I	≤4	S
Cefaxitin	16	I	≤2	S	≥64	R	16	I	16	I	≤2^*∗*^	R^*∗*^
Cefoxitin	≤4	S	≤4	S	≥64	R	≥64	R	≥64	R	8	R^*∗*^
Ceftazidime	≤1	S	≤1	S	4	R^*∗*^	4	S	8	S	≤1	S
Ceftriaxone	≤1	S	≤1	S	32	R	≥64	R	32	I	≤1	S
Cefepime	≤1	S	≤1	S	2	I^*∗*^	2	S	2	S	≤1	S
Amikacin	≤2	S	≤2	S	4	I^*∗*^	≤2	S	≤2	S	≤2	S
Ciprofloxacin	≤0.25	S	≤0.25	S	0.5	S	≤0.25	S	≤0.25	S	≤0.25	S
Tigecycline	≤0.5	S	≤0.5	S	≥8	R	≥8	R	≥8	R	≤0.5	S
Nitrofurantoin	≤16	S	64	I	≥512	R	≥512	R	≥512	R	64	I
Trimethoprim/sulfamethoxazole	≥320	R	≤20	S	160	R	160	R	320	R	≤20	S

S = susceptible; I = intermediate; R = resistance; ^*∗*^AES modified; MIC = minimum inhibition concentration; Inter = interpretation.

**Table 4:** Antibiotics susceptibility against selected Gram-positive bacteria.

Antimicrobial agents	Bacteria											
	*Staphylococcus aureus*		*Streptococcus pneumonia*		*Staphylococcus vitulinus*		*Streptococcus thoraltensis*		*Aerococcus viridians*		*Kocuria rhizophila*	
	MIC	Inter	MIC	Inter	MIC	Inter	MIC	Inter	MIC	Inter	MIC	Inter
Cefoxitin screen	POS	+	NEG	—	POS	+	POS	+	NEG	—	NEG	—
Benzylpenicillin	≥0.5	R	≥0.5	R	≥0.5	R	≥0.5	R	≤0.5	S	≤0.5	S
Oxacillin	≥4	R	≤0.5	S	≥4	R	≥4	R	≤0.5	S	≥4	R
Inducible clindamycin resistance	NEG	—	NEG	—	NEG	—	NEG	—	NEG	—	NEG	—
Erythromycin	4	R	4	R	≥8	R	≥8	R	≤0.5	S	≤0.5	S
Clindamycin	0.5	R	≤0.5	S	≥8	R	≥8	R	≤0.5	S	≤0.5	S
Linezolid	4	S	4	S	≥8	R	≥8	R	4	S	4	S
Teicoplanin	4	S	4	S	≥32	R	≥32	R	4	S	≤0.5	S
Vancomycin	2	S	2	S	≥32	R	≥32	R	2	S	1	S
Tetracycline	≤1	S	≤1	S	≥16	R	2	R^*∗*^	≤1	S	4	R
Fosfomycin	16	S	16	S	≥128	R	≥128	R	16	S	16	S
Nitrofurantoin	64	I	≤16	S	≥512	R	≤16	S	≤16	S	≤16	S
Fusidic acid	8	I	≤0.5	S	≥32	R	≥32	R	≤0.5	S	≤0.5	S
Rifampicin	4	R	≤0.5	S	≥32	R	≥32	R	≤0.5	S	≤0.5	S
Trimethoprim/sulfamethoxazole	≥320	R	≤10	S	80	R	≤10	S	≤10	S	≤10	S

S = susceptible; I = intermediate; R = resistance; ^*∗*^AES modified; MIC = minimum inhibition concentration; Inter = interpretation.
